# Long-term follow-up after rituximab plus bendamustine in a patient with relapsed or refractory hairy cell leukemia variant: A case report

**DOI:** 10.1097/MD.0000000000024457

**Published:** 2021-02-05

**Authors:** Naoto Imoto, Daisuke Koyama, Isamu Sugiura, Shingo Kurahashi

**Affiliations:** a Division of Hematology and Oncology, Toyohashi Municipal Hospital, Aichi; b Division of Hematology, Nagano Red Cross Hospital, Nagano, Japan.

**Keywords:** hairy cell leukemia variant, long-term follow-up, rituximab plus bendamustine

## Abstract

**Introduction:**

Hairy cell leukemia variant (HCL-v) is a rare lymphoproliferative disorder regarded as a splenic B-cell lymphoma/leukemia, unclassifiable tumor in the 2017 World Health Organization classification of lymphoid tumors. The prognosis of HCL-v is much worse than that of classical hairy cell leukemia and there is no consensus regarding the optimal treatment strategy for HCL-v. For patients with indolent lymphoma, rituximab plus bendamustine (RB) has proven effective in several clinical trials. Thus, RB is expected to be a treatment option for patients with HCL-v, but there have been few reports of its use in these patients.

**Patient concerns:**

A 64-year-old man presented with leukocytosis and abnormal lymphocytes in peripheral blood in a medical examination. Computed tomography revealed mild splenomegaly, but no lymph node enlargement.

**Diagnosis:**

The patient was initially diagnosed with low-grade B-cell lymphoma. After he experienced a second relapse, his clinical data were reviewed again; subsequently, he was diagnosed with HCL-v on the basis of clinical presentation, flow cytometry findings, and cytogenetic abnormalities.

**Interventions:**

The patient was first treated with the cyclophosphamide, doxorubicin, vincristine, and prednisone (CHOP) regimen. After the regimen was ineffective, he received six cycles of RB. After relapse, the patients received an additional six cycles of RB.

**Outcomes:**

The patients exhibited a slight reduction of the abnormal lymphocyte level but insufficient therapeutic efficacy during CHOP therapy. After the first cycle of RB, the patient exhibited an immediate response with the absence of minimal residual disease. He remained relapse-free for approximately 67 months. After a second relapse, complete response was again achieved with the absence of minimal residual disease following RB re-administration. He remained relapse-free for approximately 29 months after the second RB.

**Conclusion:**

RB could be a treatment option for patients with relapsed or refractory HCL-v. Further research is needed to establish the optimal treatment regimen for patients of HCL-v.

## 1 Introduction

Hairy cell leukemia variant (HCL-v) is a rare B-cell chronic lymphoproliferative disorder regarded as a splenic B-cell lymphoma/leukemia, unclassifiable tumor in the 2017 World Health Organization classification; it is now considered to be distinct from classical hairy cell leukemia (HCL-c). Most patients with HCL-v exhibit poor responses or resistance to standard treatments for HCL-c.^[1]^ To the best of our knowledge, there have been few reports regarding the effect of combined chemotherapy for HCL-v, except a regimen involving rituximab and cladribine (Cd).^[2]^

Bendamustine is an alkylating agent that is widely used for the treatment of low-grade lymphoma; it is also reportedly effective for the treatment of HCL-c when used in combination with rituximab.^[3]^ Among patients with newly diagnosed HCL-v, three were reported to achieve complete response (CR) following therapy with rituximab plus bendamustine (RB).^[4]^ However, there have been no reports of RB treatment for patients with relapsed or refractory HCL-v, or long-term follow-up after such treatment. Here, we describe a 64-year-old man who was initially diagnosed with low-grade B-cell lymphoma and later diagnosed with HCL-v. His disease was refractory to CHOP; thus, he received RB and has been followed-up for approximately 9 years in our hospital.

## 2 Case report

A 64-year-old man presented to our Department of Hematology due to the presence of leukocytosis in medical examination. He was asymptomatic and physical examinations revealed no abnormalities. He had no underlying diseases, notable medical history, or relevant family history. Initial complete blood count analysis showed a white blood cell count of 13.0 × 10^9^/L (normal range, 3.6–9.6 × 10^9^/L), 36.6% neutrophils and 56.7% lymphocytes, hemoglobin level of 13.8 g/dL (normal range, 13.2–17.2 g/dL), and platelet count of 159 × 10^9^/L (normal range, 148–339 × 10^9^/L). Blood chemistry revealed no abnormalities involving lactate dehydrogenase and soluble interleukin-2 receptor levels. Computed tomography findings showed mild splenomegaly, but no lymph node enlargement. Bone marrow aspiration was performed; the results demonstrated 23% abnormal lymphocytes with prominent nucleoli. Flow cytometry (FCM) revealed light-chain restricted B cells that were strongly positive for CD19, CD20, and CD22; positive for CD11c, κ-chain, and FMC7; and negative for CD5, CD23, CD10, and CD25. Cytogenetic analyses revealed the following abnormalities: 45, XY, der (8)t (8;13) (q24: q11), ins (8;?) (q24;?), del (11), and -13[1/20].

Accordingly, the patient was diagnosed with low-grade B-cell lymphoma. No further diagnosis could be made at that time. He received treatment with the CHOP regimen because his lymphocyte count was >5.0 × 10^9^/L. Rituximab treatment was initially avoided because of the potential for strong infusion reactions due to spleen and peripheral blood lesions. Subsequently, abnormal lymphocyte counts slightly decreased and ≥30% of the white blood cell count comprised abnormal lymphocytes; thus, the CHOP regimen was determined to be insufficient after 3 weeks of treatment. The patient then began RB therapy (day 1: rituximab 375 mg/m^2^; days 2–3: bendamustine 90 mg/m^2^). His abnormal lymphocyte count rapidly decreased, such that no abnormal lymphocytes were present in peripheral blood after 1 week of RB. After 3 weeks of RB, bone marrow smear preparations showed no abnormalities; pathologic findings, FCM, and cytogenetic analyses revealed the absence of minimal residual disease (MRD). The treatment was well tolerated and the patient completed six planned cycles of the RB regimen. The only Grade ≥3 adverse event (according to the Common Terminology Criteria for Adverse Events, Version 4.0) was lymphocytopenia. At 8 months after initiation of RB, the patient exhibited CR consistent with the 2015 European Society for Medical Oncology guidelines,^[5]^ as indicated by absence of splenomegaly in computed tomography and blood count examinations. After treatment, the absence of MRD in peripheral blood was routinely checked by FCM. At 39 months after initial chemotherapy treatment, the patient did not exhibit MRD. However, at 42 months, κ-chain restricted, CD20 and CD11c double-positive cells were detected at a rate of 0.6% in peripheral blood. Nonetheless, the proportion of cells indicative of MRD remained below 2% for an extended period.

At 69 months after initial chemotherapy treatment, abnormal lymphocytes were countable in peripheral blood smears. Complete blood count analyses showed a white blood cell count of 10.6 × 10^9^/L (30.0% abnormal lymphocytes), hemoglobin level of 13.6 g/dL, and platelet count of 118 × 10^9^/L, which met the criteria for relapse. Blood chemistry showed an elevated soluble interleukin-2 receptor level (964 U/mL; normal range, 145–519 U/mL). Mild splenomegaly and no enlarged lymphocytes were observed. Bone marrow aspiration showed 60% abnormal lymphocytes, similar to the level at initial diagnosis. FCM also showed comparable findings, including light-chain restricted B cells that expressed CD19, CD20, κ-chain, and FMC7, but did not express CD5, CD23, CD10, or CD25. Cytogenetic analyses showed the following abnormalities: 44, XY, der(7)t (7;14)(q11.2:q11.2), der(8)t (8;13) (q24:q11), ins (8;?)(q24;?), del(11)(q?), -13, and -14[6/20]. Pathologic findings were negative for TRAP, Annexin A1, and DBA44. BRAF and MyD88 both exhibited wild-type mutation statuses. Watchful waiting was first implemented due to the patient's asymptomatic disease. Two months later, chemotherapy was initiated because the patient's platelet count was below 100 × 10^9^/L and his splenomegaly had gradually progressed (ie, monthly). He received further RB treatment; abnormal lymphocytes disappeared after 1 cycle. After 3 cycles, FCM of peripheral blood showed absence of MRD. The patient completed 6 planned cycles of RB. At 79 months after initial chemotherapy treatment, computed tomography revealed absence of splenomegaly, indicative of CR.

**Figure 1:**
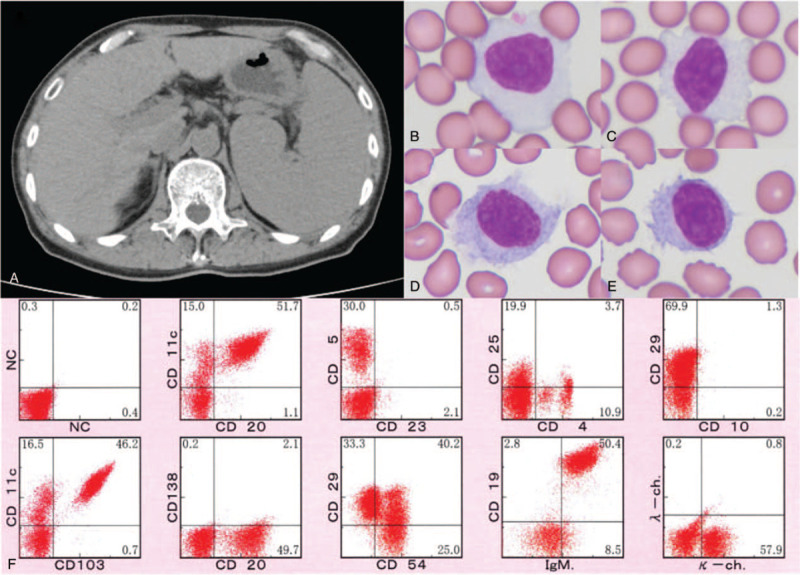
Imaging and laboratory findings during the second relapse. (A) Computed tomography shows splenomegaly. (B, C) Instrument-dried peripheral blood shows atypical cells with abundant cytoplasm. (D, E) Naturally dried peripheral blood shows villous cells with prominent nucleoli. (F) Flow cytometry analysis of peripheral blood after rituximab treatment.

**Figure 2:**
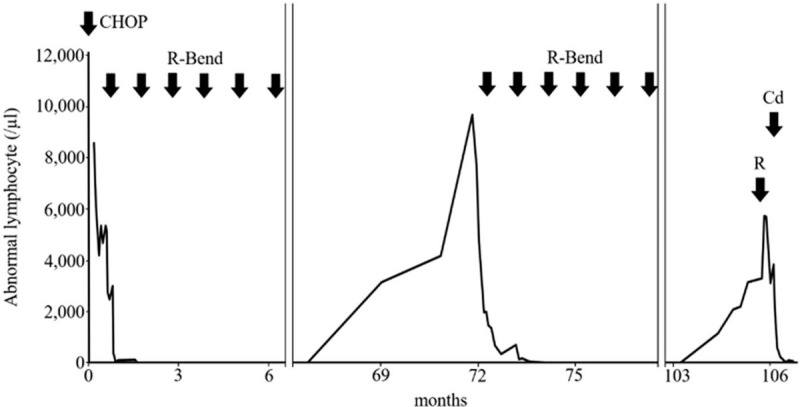
Courses of chemotherapies and changes in abnormal lymphocyte counts beginning after initial treatment with CHOP regimen. Abnormal lymphocyte counts are first visible shortly after initiation of chemotherapy. Bend = bendamustine, Cd = cladribine, R = rituximab.

At 104 months, abnormal lymphocytes were again detected in peripheral blood smear with leukocytosis, which met the criteria for a second relapse. Blood chemistry showed an elevated soluble interleukin-2 receptor level (4440 U/mL). Mild splenomegaly and no enlarged lymphocytes were observed (Fig. 1A). Bone marrow aspiration showed 15.7% abnormal lymphocytes, which was a similar finding to that of the first attachment (ie, small- to medium-sized irregular edges, with occasional clear nucleoli). FCM and cytogenetic analyses also showed abnormalities similar to those detected during the first relapse. Because the patient's abnormal lymphocyte count gradually increased (ie, weekly) and his platelet count decreased to 83 × 10^9^/L, he received rituximab monotherapy; however, this did not reduce the abnormal lymphocyte count. Thus, differential diagnosis was repeated. The atypical cells had prominent nucleoli with condensed chromatin; most also had abundant cytoplasm, as detected in instrument-dried peripheral blood, and omnidirectional hairy projections, as detected in naturally dried peripheral blood (Fig. 1B–E). Because of limitations regarding FCM antibody reagents in our hospital, FCM including CD103 was performed by a third-party laboratory. The findings were strongly positive for CD103, CD19, CD20, CD11c, and IgM; positive for κ-chain, CD29, and CD54; and negative for CD5, CD23, CD10, and CD25 (Fig. 1F). Fluorescent in situ hybridization analysis of bone marrow was performed including del (17q), del (11p), t (11;14), t (14;18), and del (13); only del (11p) was observed in 30% of cells in the specimens. Taken together, the findings supported a diagnosis of HCL-v. The patient was treated with Cd and achieved partial response (Fig. 2). The patient provided written informed consent for publication of this report.

## 3 Discussion

There is no consensus regarding the optimal treatment strategy for patients with HCL-v. Various treatment that is effective for HCL-c results in partial or no response in patients with HCL-v; remission is typically shorter in patients with HCL-v than in patients with HCL-c. Among 39 patients in six studies, the overall response rate to Cd treatment was 44%, with 8% CR.^[6–11]^ This contrasts with the good CR rates in patients with HCL-c, in whom overall CR rates >80% can be obtained with a single cycle of Cd treatment.^[2]^ The 5-year overall survival of patients with HCL-v was reportedly 57%,^[12]^ which was worse than the rate for patients with HCL-c (>95%).^[13]^ Another regimen including interferon, pentostatin, and fludarabine was also reported to elicit resistance in patients with HCL-v.^[2]^ The findings in this report suggest that RB might be effective for the treatment of patients with HCL-v.

Thus far, rituximab plus Cd has been reported to achieve the best treatment outcome for patients with HCL-v. Kreitman et al reported that a regimen of Cd immediately followed by rituximab achieved a CR rate of approximately 90% (n = 10) in patients with HCL-v; eight of the nine patients remained MRD-free at 12 to 48 months of follow-up.^[14]^ Chihara et al. reported that a regimen of Cd followed by rituximab for patients with relapsed HCL-v yielded a CR rate of 86% and 5-year event-free survival of 64% (n = 7) over a median follow-up duration of 60 months.^[15]^ Several case reports have also shown that HCL-v can be successfully treated by rituximab monotherapy. Robak described six patients who received rituximab monotherapy; 3 achieved CR and 3 achieved partial response.^[2]^ However, the response durations in that report were all within 24 months. Among other chemotherapies that have been proven effective for indolent B-cell lymphoma, little is known regarding their efficacy in the treatment of hairy cell leukemia. For patients with HCL-c, the RB regimen is an expected treatment strategy. One pilot trial showed a 100% overall response rate and a 67% CR rate following treatment with RB (rituximab 375 mg/m^2^ days 1 and 15, plus bendamustine 90 mg/m^2^ days 1 and 2, for 6 cycles at 4-week intervals) for six patients with HCL-c who had received at least 3 prior therapies.^[3]^ Regarding patients with HCL-v, Andrea et al. described 3 patients with treatment-naïve, TP53-unmutated HCL-v who were treated with RB. All patients achieved CR without MRD, which indicated that RB is an effective first-line treatment for patients with HCL-v.^[4]^ However, the median follow-up period was 19 months in that report; prior to our report, there have been no descriptions of long-term follow-up for patients with relapsed or refractory HCL-v who received RB treatment. We presume that a clinical trial is warranted to determine the long-term effects of RB treatment in patients with HCL-v.

With respect to diagnosis, it is difficult to distinguish HCL-c, HCL-v, and splenic marginal B-cell lymphoma. We ruled out a diagnosis of HCL-c for our patient because he did not exhibit mutations in BRAF V600E; moreover, he had negative findings regarding CD25, TRAP, and Annexin A1.^[16,17]^ We also ruled out splenic marginal B-cell lymphoma because the patient's abnormal lymphocytes had prominent nucleoli with omnidirectional villi; positive CD103 findings; and negative CD10 and CD23 findings.^[17,18]^ The del (11p) abnormality also supported a diagnosis of HCL-v, rather than splenic marginal B-cell lymphoma, based on prior findings.^[19,20]^

A limitation of this report is that the patient might have remained relapse-free mainly due to the slow course of his disease, rather than the effect of RB. Matutes et al. reported the natural course of disease in 52 patients with HCL-v; they found that the clinical course of HCL-v was chronic, with a long lymphocyte doubling time; however, the disease was worse than HCL-c. The overall median survival for patients with HCL-v was 9 years; 15% were alive 17 years after diagnosis.^[11]^ However, we presume that RB had an important effect in our patient because he achieved absence of MRD in bone marrow after the first cycle of RB; this indicates that RB had a remarkable treatment effect, relative to the initial CHOP regimen. Furthermore, his abnormal lymphocyte counts and splenomegaly progressed over months to weeks, which were indicative of a relatively rapid course of disease. Notably, we expect the patient's disease to progress more quickly than typical HCL-v.

In conclusion, our findings suggest that RB might be a treatment option for patients with relapsed or refractory HCL-v. Although HCL-v is a rare disease and it is difficult to compare outcomes among multiple chemotherapies, further clinical studies are warranted to determine the best treatment strategy for patients with HCL-v.

## Acknowledgments

We thank Ryan Chastain-Gross, Ph.D., from Edanz Group (https://en-author-services.edanzgroup.com/ac) for editing a draft of this manuscript.

## Author contributions

**Data curation:** Naoto Imoto, Shingo Kurahashi.

**Investigation:** Naoto Imoto, Daisuke Koyama, Isamu Sugiura.

**Project administration:** Naoto Imoto.

**Resources:** Daisuke Koyama, Isamu Sugiura.

**Supervision:** Shingo Kurahashi.

**Validation:** Daisuke Koyama, Isamu Sugiura, Shingo Kurahashi.

**Visualization:** Naoto Imoto.

**Writing – original draft:** Naoto Imoto.

**Writing – review & editing:** Naoto Imoto.
